# A sustainable artificial-intelligence-augmented digital care pathway for epilepsy: Automating seizure tracking based on electroencephalogram data using artificial intelligence

**DOI:** 10.1177/20552076241287356

**Published:** 2024-10-07

**Authors:** Pantea Keikhosrokiani, Minna Isomursu, Johanna Uusimaa, Jukka Kortelainen

**Affiliations:** 1 Empirical Software Engineering in Software, Systems, and Services, 6370 University of Oulu, Oulu, Finland; 2 Research Unit of Health Sciences and Technology, 6370 University of Oulu, Oulu, Finland; 3 Research Unit of Clinical Medicine and Medical Research Center, 60664 Oulu University Hospital, 6370 University of Oulu, Oulu, Finland; 4 Neurocenter, Neurology, 60664 Oulu University Hospital, Oulu, Finland; 5 Center for Machine Vision and Signal Analysis, 6370 University of Oulu, Oulu, Finland; 6 Cerenion Ltd, Oulu, Finland

**Keywords:** Artificial intelligence, electroencephalography, epilepsy, digital care pathway, seizure tracking, machine learning, sustainability

## Abstract

**Objective:**

Scalp electroencephalograms (EEGs) are critical for neurological evaluations, particularly in epilepsy, yet they demand specialized expertise that is often lacking in many regions. Artificial intelligence (AI) offers potential solutions to this gap. While existing AI models address certain aspects of EEG analysis, a fully automated system for routine EEG interpretation is required for effective epilepsy management and healthcare professionals' decision-making. This study aims to develop an AI-augmented model for automating EEG seizure tracking, thereby supporting a sustainable digital care pathway for epilepsy (DCPE). The goal is to improve patient monitoring, facilitate collaborative decision-making, ensure timely medication adherence, and promote patient compliance.

**Method:**

The study proposes an AI-augmented framework using machine learning, focusing on quantitative analysis of EEG data to automate DCPE. A focus group discussion was conducted with healthcare professionals to find the problem of the current digital care pathway and assess the feasibility, usability, and sustainability of the AI-augmented system in the digital care pathway.

**Results:**

The study found that a combination of random forest with principal component analysis and support vector machines with KBest feature selection achieved high accuracy rates of 96.52% and 95.28%, respectively. Additionally, the convolutional neural networks model outperformed other deep learning algorithms with an accuracy of 97.65%. The focus group discussion revealed that automating the diagnostic process in digital care pathway could reduce the time needed to diagnose epilepsy. However, the sustainability of the AI-integrated framework depends on factors such as technological infrastructure, skilled personnel, training programs, patient digital literacy, financial resources, and regulatory compliance.

**Conclusion:**

The proposed AI-augmented system could enhance epilepsy management by optimizing seizure tracking accuracy, improving monitoring and timely interventions, facilitating collaborative decision-making, and promoting patient-centered care, thereby making the digital care pathway more sustainable.

## Introduction

### Background

Epilepsy is one of the most common neurological disorders that are characterized by recurrent and unprovoked seizures.^1,2^ These seizures are caused by abnormal electrical activity in the brain, resulting in a wide range of symptoms and manifestations.^3^ Epilepsy can have a significant impact on an individual's quality of life, affecting physical, cognitive, and emotional aspects. Therefore, it is crucial to understand epilepsy, including its causes, diagnosis, and treatment options for effective management and support for individuals living with this condition.

An accurate and timely diagnosis of epilepsy is essential in digital care pathway for providing appropriate treatment and care to individuals with this neurological disorder. Electroencephalogram (EEG) has become crucial in the diagnosis and evaluation of epilepsy. EEG records the electrical activity of the brain, providing valuable insights into abnormal patterns and signatures associated with epilepsy seizures.^4^ Analyzing EEG data enables healthcare professionals to identify epileptic activity, determine seizure types, and locate the source of abnormal brain activity. The interpretation and diagnostic accuracy of EEG data have been further enhanced by using advanced computational algorithms and machine learning techniques.^5^

*Problem identification and objectives*. Clinicians often face challenges with diagnosing and managing seizures for epilepsy. Although machine learning models are proposed for automating seizure tracking and seizure classification into focal and nonfocal seizures for individuals with epilepsy, there is limited evidence on the adoption of this kind of technology in clinical practice. In order to understand the limitations of automating seizure tracking for epilepsy, healthcare professionals’ opinion and insight are very crucial.^6^ Patients with epilepsy have an unpredictable seizure frequency, which affects their quality of life. Predicting seizures or at least identifying them once they start to manifest clinically and acting quickly is beneficial. There are available seizure tracking systems on the market and have been studied, but user preferences and the true requirement are not well understood.^7,8^ Therefore, a focus group discussion was conducted for problem identification of the current digital care pathway for epilepsy (DCPE) and requirement analysis of integrating seizure tracking system into it. The details of this focus group discussion are added in the methodology section.

The focus group discussion emphasized that various advanced ECGs have been tracking cardiac issues for decades, whereas there are no similar techniques for EEG signal monitoring. Determining the kind of seizure is essential for selecting the right treatment, but a further issue is anticipating the patient's reaction to the drug. Current medications only manage partial seizures in around one-third of epileptics, which emphasizes the need for more potent therapies.^9^ Furthermore, the healthcare professionals highlighted that “currently, the time taken to diagnose epilepsy is too long, and there is a need to minimize this duration. The patient would be seriously at danger as the seizure may go on longer.” In focus group discussions, the need for sophisticated wearable wireless EEG-based seizure detection tools was identified. Rapid diagnosis, successful treatment, and economical technology integration were prioritized. However, verifying the viability and dependability of the technology is necessary before deploying a large-scale sensor-based EEG seizure monitoring system, particularly with AI integration. The cost-effectiveness of the solution suggests that resource distribution and budget will be important factors. It was proposed that the patient-centric approach can be used to improve DCPE even further.

The summary of the extracted themes is shown in Table 1. The results indicate the need of integrating remote monitoring system and seizure detection in the current DCPE. The use of existing datasets was suggested for the training of different algorithms to enhance the system accuracy and quality before the final implementation. Therefore, this study focused on the development and comparison of different algorithms using existing epilepsy seizure datasets to gain a thorough understanding of various algorithms before developing a novel model for seizure detection.

**Table 1.** Summary of themes, subthemes, and codes for requirement analysis based on focus group discussion.

Theme	Subtheme	Codes extracted from quotations
Healthcare technology innovation	Remote monitoring	Real-time patient assessment, home monitoring, intensive care unit, continuous monitoring, patient care
	Seizure tracking	EEG monitoring, artificial intelligence, algorithm for seizure tracking
Data and technology integration	Use of existing data	Leveraging existing datasets, training algorithms, enhance system accuracy
	Ubiquitous health (uHealth)	Universal and accessible health monitoring
Patient-centered care	Sensitivity to patient information	Patient privacy, ethics, concerns
	Tailoring to patient needs	Customizing the system, meet patient needs, careful planning
Technical challenges and solutions	Sensor technology	Challenges, selections of sensors, implementing health technology
	System feasibility and development	Testing system feasibility, integrating new technology into the healthcare pathway
Strategic planning and implementation	Involvement of external partners	Defining goals, careful planning, strategies
		Priorities, strategic phased, specific patient groups, hospital environments
Collaboration and partnership	Involvement of external partners	Industries for sensors, technology development, multistakeholder approach

EEG: electroencephalogram.

Furthermore, the results highlighted the sensitivity of patient information and solutions for keeping patient privacy. It was also noted that the system must be aligned with the patients’ need specially for selecting the type of sensors for EEG-based seizure detection. Thus, some external industries were recommended for the selection of the validated wearables. Finally, the requirements were prioritized to be used for the adults in intensive care unit (ICU) department first, and in case of successful development, the system can be extended for patients of different age categories as well as home monitoring system.

In the current DCPE a digital platform with mobile applications is developed to empower the patient's personalization and reduce visiting to the hospitals. However, a platform for shared decision-making and the patient-centric care pathways with AI-augmented elements to automate the process of diagnosis, control, adherences, and care are not yet developed for the current care pathway. Especially, the seizure detection and classification process are done manually based on the clinicians’ observation from the EEG and ECG signals provided by the electrocardiographic monitoring machines.

Employing the power of EEG data analysis can enhance early detection and diagnosis of epilepsy, leading to more precise treatment strategies and improved outcomes for individuals living with this condition.^10^ EEG-based seizure detection needs be added into the epileptic digital care pathway to automate the epileptic diagnosis, treatment, and care process. Therefore, this study aims to propose an AI-augmented framework for automatic detection of seizure to monitor individuals with epilepsy, provide a platform for shared decision-making and patient-centered care, and offer timely and adequate medication adherences and compliance. Various algorithms are compared in terms of performance to finalize their suitability for designing a novel algorithm to classify seizures for individuals with epilepsy. In order to identify the issues of the current digital care pathway and assess the feasibility, usability, and sustainability of the AI-augmented system in the digital care pathway, a focus group discussion was conducted with healthcare professionals from Oulu University Hospital. Finally, a new epileptic digital care pathway is designed to automate the process of diagnosis in epilepsy. The remainder of the paper focused on the proposed methodology, results, and analysis, followed by discussion and conclusions.

## Literature review

### DCPE

Digital care pathways in epilepsy refer to the use of digital technologies and platforms to enhance patient care, increase accessibility to healthcare services, and improve overall outcomes for individuals living with epilepsy.^11–14^ The objective of digital care pathways is to integrate different healthcare data sources, such as electronic health records (EHR), wearable devices, and patient-reported outcomes, into a comprehensive and interoperable system. This integration can lead to more coordinated and holistic care for patients with epilepsy. Decision support systems that incorporate artificial intelligence (AI) algorithms can assist healthcare providers in making informed decisions about diagnosis, treatment, and ongoing management of epilepsy.^15^

By boosting patient participation and enhancing treatment results, digital care pathways have the potential to completely transform the way epilepsy patients receive their care. However, it is important to consider factors such as data privacy, security, and reasonable access to ensure ethical and effective implementation of these technologies in epilepsy management.^12^

Digital tools can provide educational resources, self-management tools, and personalized care plans^16^ that empower individuals with chronic diseases. These resources can help patients understand their condition better, track their symptoms, and actively participate in their treatment.^17–19^ Decision support systems that incorporate AI can assist healthcare providers in making informed decisions about diagnosis, treatment, and ongoing management of chronic conditions.^20–23^ Digital care pathways have the potential to revolutionize the delivery of care for epilepsy patients by increasing patient engagement and improving treatment outcomes. However, different aspects of sustainability in terms of economic, social, and environmental aspects need to be taken into consideration in the design and development of digital care pathway for chronic diseases such as epilepsy.^12–14^

### Remote monitoring and seizure tracking

The defining characteristic of epilepsy is epileptic seizures that occur due to abnormal and excessive electrical activity in the brain. These episodes can be unpredictable and vary in severity, duration, and manifestation. Epilepsy seizures can significantly impact an individual's daily life, relationships, and overall well-being. Hence, recognizing the nature of these seizures, their triggers, and available management strategies to provide appropriate support and care for individuals living with epilepsy are essential.^24^ However, the challenges of epileptic prediction and seizure detection based on EEG dataset remain.^25–27^

Adding remote monitoring in digital care pathways for epilepsy allows for continuous observation of patients in their natural environments, providing a more comprehensive understanding of seizure patterns compared to traditional in-hospital visits. This can lead to more timely and accurate adjustments in treatment plans, improving seizure control and quality of life for patients.^13,14,28,29^ The primary benefits of remote monitoring include improved seizure management, enhanced patient safety, and increased patient autonomy. Remote monitoring systems can alert caregivers and medical professionals to seizure events in real time, potentially reducing the response time during emergencies.^29,30^ Furthermore, continuous data collection helps in identifying triggers and patterns that may not be evident through periodic clinic visits alone.

Despite its advantages, remote monitoring faces several challenges. Privacy concerns and data security are vital, as sensitive health information is transmitted and stored digitally. Moreover, the accuracy of some devices can be affected by various factors, such as the patient's activity level or device placement. There are also issues related to the cost and accessibility of advanced monitoring technologies, which may not be affordable for all patients.

### EEG-based seizure classification for epilepsy

EEG data plays a critical role in detecting epilepsy seizures by capturing unique patterns and abnormalities associated with epileptic seizures through recording the electrical activity of the brain. Healthcare professionals analyze EEG data to identify specific changes in brainwave patterns that indicate seizure occurrence. Several key features in EEG data aid in epilepsy seizure detection, including sharp spikes, slow wave discharges, rhythmic patterns, and interictal epileptiform discharges. These abnormal patterns can be visually identified and analyzed by experienced neurologists or automated algorithms.^31,32^ Furthermore, EEG data can be used for long-term monitoring to capture infrequent or nocturnal seizures that may not be observed during routine clinical examinations. This continuous monitoring helps healthcare providers obtain a comprehensive picture of the patient's seizure activity and guides treatment decisions.^31–33^

EEG data is a valuable tool for epilepsy seizure detection. It allows for the identification of abnormal brainwave patterns associated with seizures, aids in distinguishing epileptic seizures from other events, provides long-term monitoring capabilities, and benefits from advancements in computational techniques for automated detection. By utilizing EEG data, healthcare professionals can make accurate diagnoses, tailor treatment plans, and improve the management of epilepsy for individuals affected by this condition. Machine learning techniques have shown promise in detecting epileptic seizures by analyzing patterns and features in EEG signals. They can assist in the automatic detection and classification of epileptic seizures by extracting relevant features from EEG signals using various time-domain, frequency-domain, and time-frequency analysis techniques.^33^

For real-time seizure detection, low-latency algorithms and efficient feature extraction methods are crucial. The computational complexity of the model should be considered for deployment on embedded systems or portable devices. Commonly used techniques for seizure detection and classification include spectral power, wavelet coefficients, statistical measures, and entropy measures. Once these features are extracted from EEG samples, machine learning algorithms such as support vector machines (SVM), artificial neural networks, random forests (RF), and k-nearest neighbors (k-NN) are used to distinguish between seizure and nonseizure patterns using labeled data.^10^ Transfer learning techniques can be applied to leverage pretrained models on larger datasets and adapt them to the specific task of seizure detection. Deep learning models like convolutional neural networks (CNN) or recurrent neural network (RNN) have shown promising results in automatic seizure detection by directly processing raw EEG signals.^34,35^

## Methods

This study aims to propose an AI-augmented framework for automating seizure tracking in the current DCPE using machine learning. For this reason, a mixed-method study was conducted in which a quantitative approach was utilized based on the EEG dataset collected in Department of Epileptology, University Hospital Bonn. Furthermore, a series of focus group discussions with medical professionals chosen from Oulu University Hospital were held from May to December 2023 in order to identify issues with the current DCPE and assess the viability, usability, and long-term viability of the AI-augmented system. However, this study concentrates on one of the discussions related to automating seizure tracking in the current DCPE in Oulu, Finland.

### Focus group discussion

**Figure 1.**
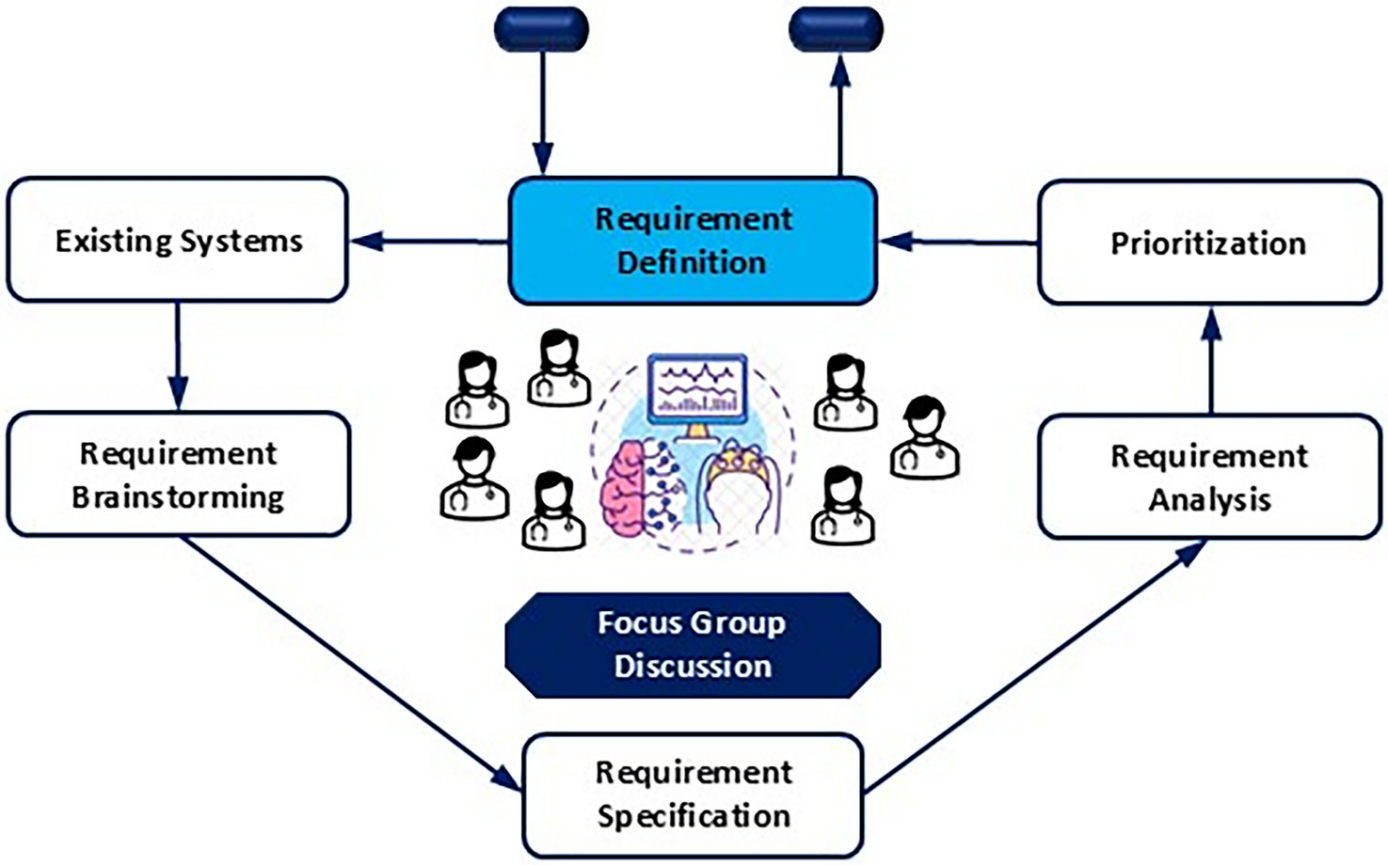
Focus group discussion for problem identification and requirement analysis of automating seizure tracking in digital care pathway for epilepsy.

As shown in Figure 1, focus group discussion was conducted for problem identification of the current DCPE and requirement analysis of integrating seizure tracking system into it. The focus group discussion involved a few steps consisting of the comparison of existing systems, brainstorming, requirements specification, analysis, and finally requirement prioritization. Purposive sampling was utilized to select a healthcare professional team from Oulu University Hospital, Finland for this study, which involves neurologist, digital health experts, pediatric neurologists, clinical neurophysiologist, expert in EEG signal processing (from University of Oulu and Industry), and senior physician. Participants were approached via email and telephone. A semistructured interview was conducted using a flexible open-ended questionnaire, which was pilot-tested. This round of focus group was arranged to last 45–90 min with seven participants. A qualitative content analysis was used for collected data, the findings were coded, themes were identified, and an interpretation was provided in a narrative format. Data saturation was achieved to extract proper conclusions.

Many studies have focused on the technical aspects of automated seizure tracking,^8,21,25,27,29^ such as algorithm performance, data preprocessing, and signal analysis. However, the healthcare professionals’ perspectives were not considered on those studies while they are equally crucial for several reasons such as understanding user need, usability, feasibility, and sustainability of the proposed AI-augmented seizure management system in the digital care pathway.

### Proposed AI-augmented framework for seizure tracking in digital care pathway

**Figure 2.**
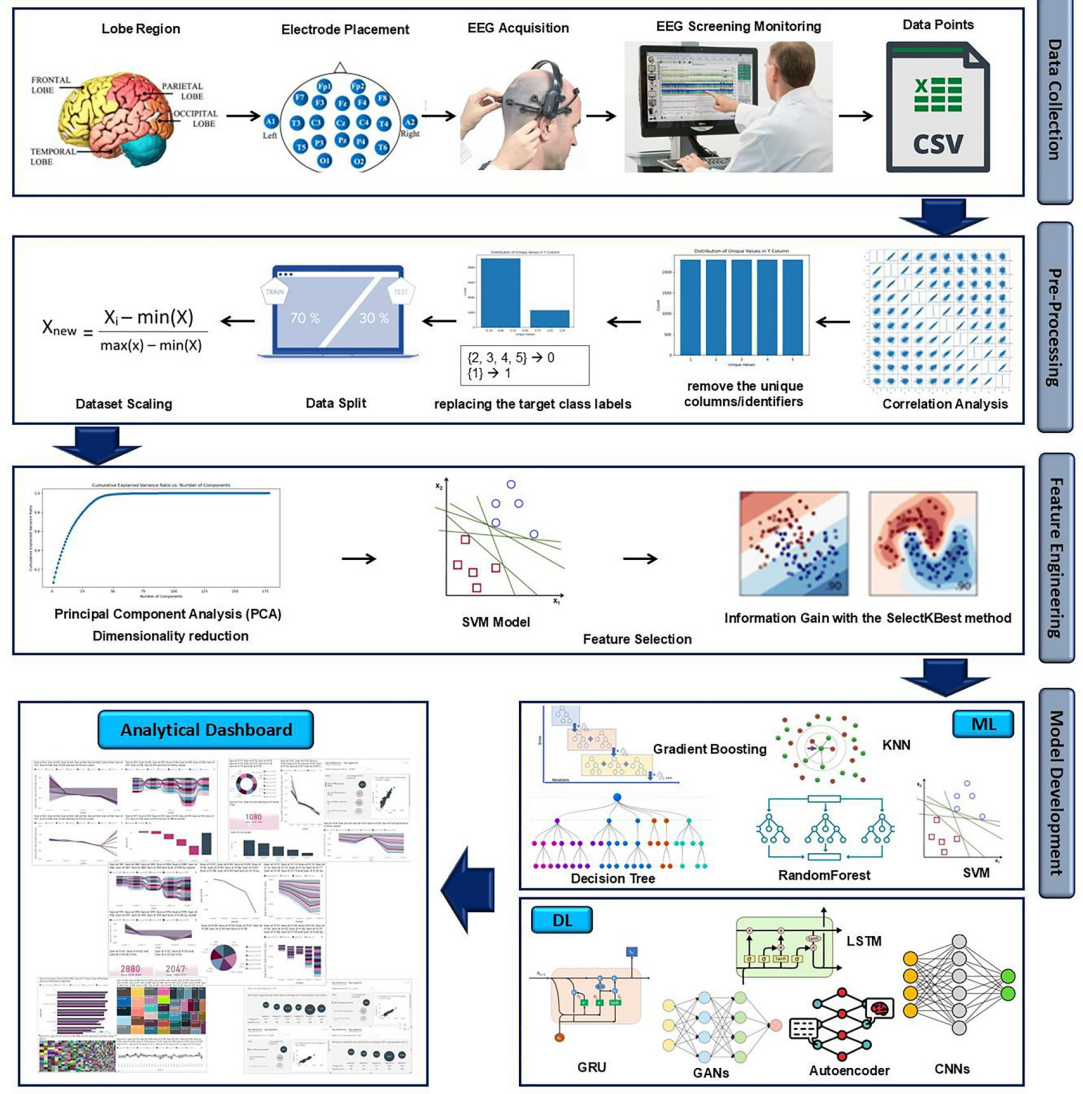
The proposed AI-augmented framework for integrating EEG-based seizure tracking into DCPE.

AI: artificial intelligence; EEG: electroencephalogram; DCPE: digital care pathway for epilepsy.

Accurate classification of epileptic seizures plays a crucial role in the diagnosis and management of epilepsy, a neurological disorder affecting millions of individuals worldwide. Recent advancements in machine learning and AI have further improved the capabilities of EEG data analysis for seizure detection. Algorithms can be trained to automatically detect and classify seizure events, providing real-time alerts to healthcare providers or individuals themselves. Therefore, this study proposed an AI-augmented framework (Figure 2) for EEG-based seizure classification which can be added into epileptic digital care pathway. The proposed model can provide better understanding to the healthcare professionals for detecting epileptic seizure. The EEG dataset collected from wearable caps needs to be preprocessed first before developing the predictive model. Furthermore, feature engineering including scaling is required to achieve a consistent arrangement of the features and prevent certain features from dominating others due to their original scale.

### Data collection and prep-processing

The EEG data acquisition or data collection step involves employing electrodes positioned on the scalp to measure and record the electrical activity of the brain. The electrodes need to be located on the patient's scalp properly and it is important to perform calibration procedures before recording the actual data. The acquired EEG dataset needs to be monitored by the experts and labeled accordingly before developing the predictive model. For this study, a published EEG dataset is used, which was collected in Department of Epileptology, University Hospital Bonn.^36^ The dataset contains recordings of brain activity from 500 subjects, each represented by 4097 data points. Each data point in the recording can be considered a feature. The dataset originally was divided into five folders, in which each folder includes 100 files corresponding to a single subject. Each file represents a 23.6 s recording of EEG activity, which is sampled into 4097 data points. To facilitate analysis, we have further divided and shuffled the data points into 23 chunks, with each chunk containing 178 data points representing 1 s of EEG activity. Additionally, each chunk is labeled with a response variable, y, indicating the category of the EEG recording. The labels range from 1 to 5, where: 1: recording of seizure activity, 2: tumor was located, 3: healthy brain area, 4: patient eyes are closed, and 5: patient eyes are open. Since this study focuses on epileptic seizure classification, binary classification is used, which includes Class 1 (epileptic seizure) and the rest of the classes are considered as 0 (nonseizure conditions).

From the dataset shape (11,500, 180), it is confirmed that it contains 11,500 instances which refers to (500 individuals * 23 s for each). Moreover, there are 180 columns where the first column Unnamed: 0 for the instance identifier, then the 178 columns for the EEG value, plus the last column for the target class y. The number of columns makes the data to be considered as high dimensional data; therefore, reducing the dataset dimensionality is required as it helps the algorithms to better learn the data and at the same time reduce its computational cost. Furthermore, the dataset has a balanced distribution of data across the different classes, namely 1, 2, 3, 4, and 5. The consistent count of 2300 for each label suggests a uniform representation of these labels within the dataset.

Since this study aims to use binary classification, the labels {2, 3, 4, 5} are reassigned as 0, and the Label 1 as it is. This approach simplifies the classification task by grouping labels into two distinct categories. By mapping Labels 1 and 0 for instances with and without epileptic seizures. This binary classification setup allows for a more focused analysis and modeling process, where the goal is to classify instances as either having an epileptic seizure (1) or not having an epileptic seizure from Label 2–5 (0).

### Feature engineering

#### Dataset scaling

In this study, the values in datasets range from −2000 to 2000, and the main aim is to scale them to a more standardized range of 0 to 1. This scaling helps in achieving consistent ranges and preventing certain features from dominating others due to their original scales. As shown in the following equation, in this study min–max scaling technique is used to transform the values proportionally to fit within the desired range. This scaling process ensures that each feature is rescaled to a common range, enabling fair comparisons, and avoiding potential bias towards features with larger magnitudes. Ultimately, scaling the data allows machine learning models to converge faster and perform optimally by effectively utilizing the features within a consistent and normalized range:

$$X_{scaled}=\frac{x-\min(x)}{\max(x)-\min(x)},$$

where *x* is the original value, min(*x*) is the minimum value in the dataset, and max(*x*) is the maximum value in the dataset.

For each feature column $x_{i}$ in the dataset,

$$X_{i,scaled}=\frac{x-\min(x_{i})}{\max(x)-\min(x_{i})}.$$

#### Dimensionality reduction and feature selection

In this step principal component analysis (PCA) is used and features are selected to reduce the dimensionality of the dataset while retaining the most valuable features. By leveraging the strengths of both techniques, this study aims to achieve a more compact and informative feature set that can enhance the accuracy and efficiency of our subsequent modeling tasks. After applying PCA to the dataset, a remarkable reduction in dimensionality is obtained. Out of the original 178 features in the dataset, only 53 features were needed to retain an impressive 99.05% of the total information. This reduction indicates that the majority of the variance in the data can be explained by this subset of features. The reduced dimensionality not only simplifies the dataset but also has potential benefits in terms of computational efficiency and model interpretability. With this condensed feature representation, analysis or modeling can be done, focusing on the most informative aspects of the data while minimizing the risk of overfitting or noise. Therefore, 53 number of features are used for further preprocessing. Moreover, the performance of the extracted PCA features is tested using SVM algorithm.

The main objective of this step is to reduce the number of features in the dataset while retaining as much information as possible. PCA is affected by scale; therefore, we need to scale the features in the data before applying PCA using the following equation. You can use standard scaling (mean = 0 and variance = 1):

$$Z_{i}=\frac{\left(x_{i}-\mu_{i}\right)}{\sigma_{i}},$$

where $\mu_{i}$ is the mean of feature *i* and $\sigma_{i}$ is the standard deviation of feature *i*.

The next step is to compute the covariance matrix, which is a square matrix giving the covariance between each pair of elements of a given random vector:

$$\sum \frac{1}{n-1}(X-\mu {)}^{T}(X-\mu ),$$

where *X* is the standardized data matrix, μ is the mean vector, *n* is the number of data points.

Then the eigenvectors and eigenvalues of the covariance matrix need to be calculated using the following equation to identify the principal components:

$$\sum \vartheta =\lambda_{\vartheta },$$

where ϑ are eigenvectors and λ are eigenvalues.

The next step focuses on selecting the principal components. In this step, we need to sort the eigenvalues in decreasing order and select the top *k* eigenvectors that correspond to the largest eigenvalues (these capture most of the variance in the dataset). In order to display the data, the original matrix *X* needs to be transformed using the selected eigenvectors to reduce the dimensions of the data as shown in the following equation:

$$X_{reduced}=X_{\vartheta_{k}},$$

where $\vartheta_{k}\;$ are the selected k eigenvectors. The transformed data $X_{reduced}$ has fewer dimensions (from 178 to 53 in your case) but retains 99.05% of the variance.

### AI model development

This study aims to integrate epilepsy tracking into the current digital care pathway, which monitors and detects seizures automatically using machine learning. An AI-augmented framework is proposed for this reason, which examined and compared different machine learning and deep learning algorithms to find how these algorithms can accurately identify epileptic seizures in digital care pathways. Therefore, as suggested by other researchers in the existing studies,^37–39^ SVM, RF, K-NN, decision tree (DT), and gradient boosting are selected as the machine learning algorithms for creating the AI model to classify epileptic seizure. Among deep learning algorithms, CNNs,^40^ using one-dimensional convolutional layer with 64 filters, a kernel size of 3, and Epoch 100, long short-term memory (LSTM)^41^ with 64 filters, timesteps (178), and Adam optimizer, autoencoder, generative adversarial networks (GANs), gated recurrent unit (GRU) are selected for this study. In order to evaluate the performance of the algorithms, metrics like precision, recall, F1-score, and area under curve-receiver operating characteristic (AUC-ROC) are utilized as these metrics help account for class imbalances and provide a comprehensive evaluation of classification models. Furthermore, GridSearch is used for algorithms that tend to overfit the data.

## Results and analysis

Tables 2 and 3 compare the performance of different algorithms. Based on the PCA feature selection, SVM achieved an accuracy of 95.77%, which indicates its effectiveness in capturing the underlying patterns in the data. RF attained an accuracy of 96.52%, demonstrating the power of ensemble learning and feature selection in improving classification performance. The accuracy for k-NN was 89.423%, indicating its limitations in handling high-dimensional data or capturing complex relationships. DT gained an accuracy of 91.21%, providing a simple yet effective approach for classification tasks. Finally, gradient boosting achieved an accuracy of 96.41%, showcasing the ability of boosting algorithms to improve performance by combining weak learners. The results show that the best match in PCA feature selection is RF since the model achieved higher accuracy and AUC-ROC score of 99.03%. This indicates that the model has performed exceptionally well in distinguishing between the positive and negative classes. This high score suggests that the model has captured the underlying patterns and relationships present in the train dataset effectively.

**Table 2.** The comparison of different machine learning models using PCA and KBest feature selection.

Algorithm	Accuracy (%)	Precision (%)	Recall (%)	F1 score (%)	AUC-ROC (%)
PCA for feature selection					
SVM	95.77	89.88	89.11	89.50	93.28
Random forest	96.52	91.52	91.26	91.39	94.65
k-NN	89.42	97.17	49.14	65.27	74.39
Decision tree	91.21	74.25	85.53	79.49	89.00
Gradient boosting	96.41	91.12	91.12	91.12	94.43
KBest feature selection					
SVM	95.28	93.5	82.52	87.06	90.52
Random forest	94.93	90.92	83.24	86.91	90.57
k-NN	94.06	94.74	74.79	83.59	86.87
Decision tree	93.07	86.84	77.51	81.91	87.26
Gradient boosting	94.90	91.43	82.52	86.75	90.28

AUC-ROC: area under curve-receiver operating characteristic; k-NN: k-nearest neighbors; PCA: principal component analysis; SVM: support vector machines.

**Table 3.** The comparison of different deep learning algorithms.

Algorithm	Accuracy (%)	MSE
SVM	95.80	
RandomForest	96.58	
k-NN	93.33	
Decision tree	93.57	
Gradient boosting	96.46	
CNNs	97.65	
LSTM	90.17	
GRU	97.59	
Autoencoder		0.0015
GANs		0.36

CNN: convolutional neural network; GAN: generative adversarial network; GRU: gated recurrent unit; k-NN: k-nearest neighbors; LSTM: long short-term memory; SVM: support vector machines.

On the other hand, for KBest feature selection, SVM shows a stable performance in compared with other algorithms. The model achieved an AUC-ROC score of 98.19%, which indicates the model has performed exceptionally well in distinguishing between the positive and negative classes during the training phase. This high score suggests that the model has captured the underlying patterns and relationships present in the train dataset effectively. The results indicate that the model has a strong ability to distinguish between positive and negative classes. However, while the score is still high, the results are still lower compared with using PCA.

Based on the results of deep learning algorithms shown in Table 2, CNN achieved impressive results of with a validation accuracy of 97.65% after 100 epochs of training. The model's low loss value of 0.0074 indicates its ability to accurately classify the EEG data. These results demonstrate the effectiveness of CNNs in capturing complex patterns and features within the EEG signals, enabling accurate binary classification of epileptic seizures. The LSTM model achieved a loss of 0.281 and an accuracy of 90.17%. This indicates that the model performed reasonably well in capturing the temporal dependencies and patterns in the EEG data for epileptic seizures classification. The relatively lower loss suggests that the model was able to minimize the discrepancy between the predicted and actual outputs. The validation loss for the autoencoder model was 0.0015, and the loss was extremely low at 0.0010. According to these findings, the model successfully recreated the input data, capturing significant patterns and characteristics. Autoencoders are effective unsupervised learning models for feature extraction and dimensionality reduction. In this study, the EEG data was effectively compressed using the autoencoder, maintaining important information with little loss. A 97.59% accuracy rate for the GRU shows how well this condensed form of LSTM captures temporal relationships. Finally, GANs achieved an mean squared error (MSE) of 0.36, illustrating the ability to generate synthetic EEG signals that closely resemble the original data distribution.

Overall, these results highlight the strengths and limitations of different machine learning and deep learning algorithms in handling EEG data for epileptic seizures classification. CNNs and RF performed exceptionally well, while LSTM showed room for improvement. The low MSE values obtained by autoencoder and GANs indicate their potential for data reconstruction and generation tasks. Furthermore, experimentation and fine-tuning of the models can help optimize their performance and potentially improve the accuracy for epileptic seizures.

## Discussion

Based on the observation during an expert meeting and focus group discussion, current epilepsy care pathways have limited AI-based features specially for remote monitoring and seizure detection. Therefore, this study aims to propose an AI-augmented framework to automate seizure tracking using EEG dataset to sustain the current digital care pathway. Different algorithms are compared in terms of performance. The results indicate that RF on PCA and SVM on KBest feature selection achieved the best accuracy of 96.52% and 95.28% respectively. Furthermore, CNN outperforms the other deep learning algorithms with the accuracy of 97.65%. The result of this study leads to the propose of a novel algorithm with higher accuracy for epileptic seizure classification, which can be integrated into the current digital care pathway. Perhaps combining CNN and RF or CNN and GRU as a hybrid model might be a good idea to yield higher accuracy. However, it is important to note that only combining two effective algorithms does not ensure improved performance. The interoperability, data type, feature extraction abilities, and other elements are critical to a hybrid model's performance. In actuality, it would be wise to experiment with various pairings and test the performance of the hybrid model on a validation dataset.

**Figure 3.**
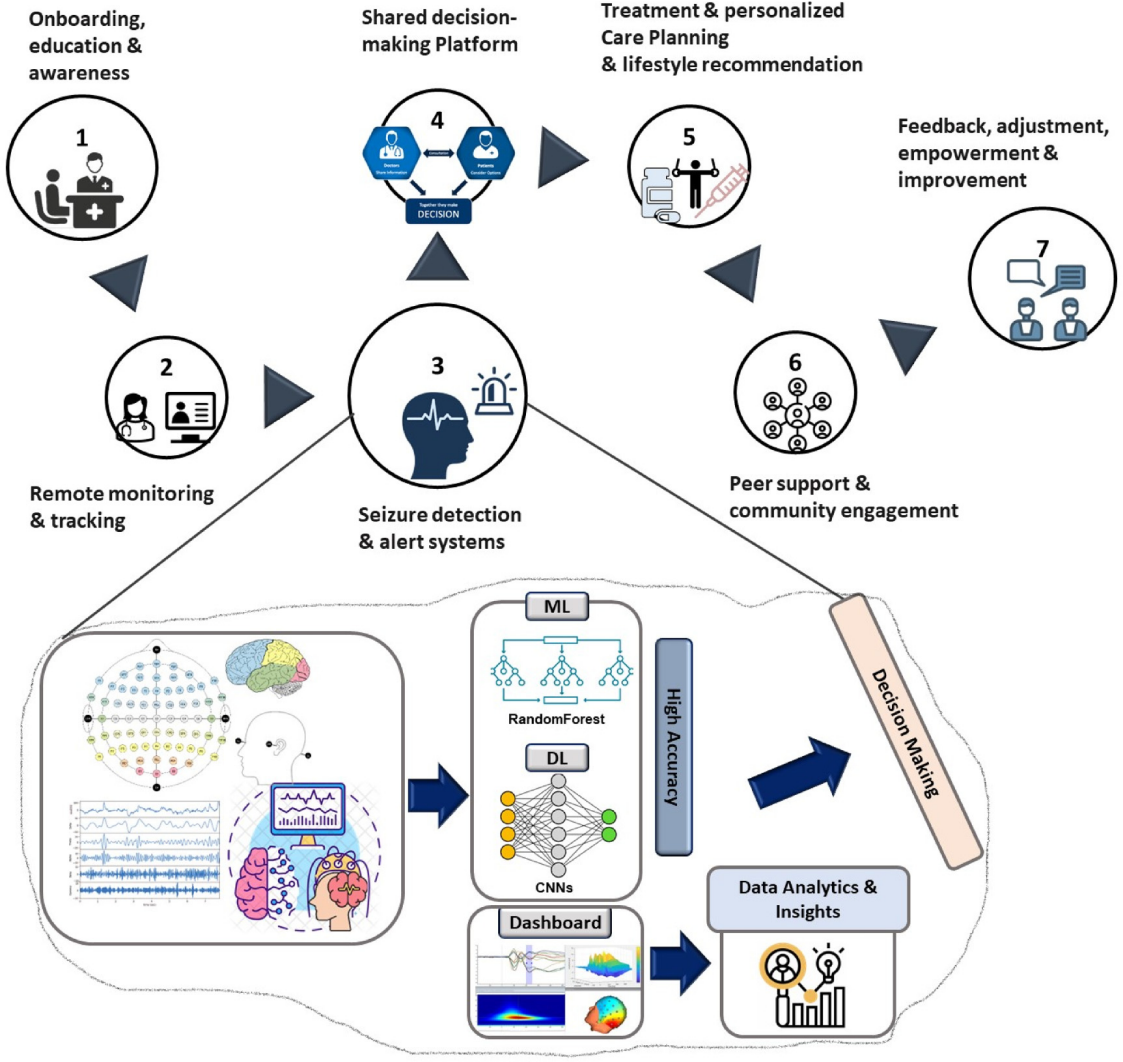
The proposed AI-augmented sustainable digital care pathway for epilepsy.

AI: artificial intelligence; EEG: electroencephalogram.

Based on the insight from the focus group discussion, a futuristic AI-integrated DCPE^27–29^ is proposed in this study (Figure 3) to automate the process of diagnosis. AI-integrated DCPE incorporates seizure detection for enhanced shared decision-making and patient-centered care. It includes various components to support individuals with epilepsy in managing their condition effectively.

### Onboarding, education, and awareness

This step mainly focuses on registering patients in digital care pathway, collecting baseline historical health records, such as previous seizure, seizure diaries, medications, and other relevant information. In addition, a digital platform needs to be developed to educate the patients and provide comprehensive information about epilepsy, its causes, symptoms, diagnosis, treatment options, and lifestyle management. This digital platform needs to incorporate interactive educational resources such as videos, infographics, metaverses, and articles to improve literacy skills and to help users understand epilepsy and related technologies better. It also needs to offer information on seizure first aid, safety precautions, and how to create a seizure management plan.

### Remote monitoring and tracking

This step requires a mobile app or web-based system with the integration of wearable devices that allows individuals to track and record their seizures, medication intake, and side effects. It enables users to record information about seizures in this stage, including the date, time, length, causes, and postseizure symptoms. It also incorporates reminders for taking medications as prescribed and offers a capability to track medication use to measure medication adherence.

### Seizure detection and alert systems

This step is required to detect seizures in real-time, including seizure detection algorithms into wearable technology or mobile applications. When a seizure is discovered, automatically notify the selected contacts (family, caretakers, or healthcare providers), providing if available the user's location. This phase of the care pathway empowers the users to manually activate notifications during crises or when they need immediate assistance.

### Shared decision-making platform

This step provides access to the analyzed data for both patients and healthcare professionals via a secure digital portal. It gives patients the option to add any notes, observations, or worries about their seizures. However, the type of shared information needs to be confirmed with the healthcare professionals.

### Treatment, personalized care planning, and lifestyle recommendation

This stage gives users the resources they need to develop personalized care plans based on the type of epilepsy, triggers, lifestyle habits, and treatment objectives.

Healthcare professionals may recommend modifying a patient's medication, dosage, or other therapies based on the results extracted from the data analysis. A collaborative treatment plan might be created as a result of patient input on their comfort level with the suggested adjustments. A personalized recommendation can be given to the patients for seizure management, medication adherence, lifestyle modifications, and stress reduction techniques.

### Peer support and community engagement

This step helps to create a community or forum online where people with epilepsy and their families may get in touch, exchange stories, and offer support to one another. It allows people to participate in discussion forums, join support groups, and access trustworthy websites that medical experts suggest. Finally, it gives users the chance to participate in conferences, webinars, or training sessions relating to epilepsy.

### Feedback, adjustment, improvement, and empowerment

This stage enables users to monitor their advancement and get encouraging feedback and reminders to stay on track. It provides a platform that continuously asks patients for comments on how the digital care pathway and seizure detection system have affected them. It makes the required changes to improve the patient experience and treatment results. In this stage, patients are empowered by some tools to actively participate in their own care and decision-making.

As summarized in Table 4, further discussion was conducted with the healthcare professionals to find the feasibility, usability, and suitability of integrating AI-augmented seizure detection in digital care pathway.

**Table 4.** Summary of themes, subthemes, and codes for focus group discussion results.

Theme	Subtheme	Codes extracted from quotations
Sustainability	Environmental sustainability	Electronic device lifecycle, energy-efficient devices, reducing the need for physical travel, carbon footprint
	Economic sustainability	Cost-effective, lower travel costs, reduced emergency room visits
	Social sustainability	Accessibility, job satisfaction, patient care, decision-making, training and support systems, enhancing user digital literacy, personalized care, equal care pathway which is available from anywhere at any time
Integration	Usability and feasibility	Interoperability, adaption to technological advancement, careful planning, collaboration, flexibility and scalability, continuous feedback, regular updates, user experience, patient accessibility, digital literacy
User-centered design	Patient support, personalized care plans	Feedback loop, accessibility features, continuous improvement, outcome measurement
	
Data security and privacy	Compliance	GDPR, security measures
	
Training and support	Availability of skilled personnel, digital literacy	Comprehensive training programs, technical support, scalability
	
Technological infrastructure	Availability, training programs	
Collaboration and partnership	Involvement of external partners	Industries for sensors, technology development, multistakeholder approach

GDPR: General Data Protection Regulation.

The finding of the focus group discussion shows that the proposed AI-augmented framework for integrating EEG-based seizure tracking make the digital care pathway more sustainable from environmental, economic, and social aspect.^13^

*Environmental sustainability.* Implementing the proposed digital care pathway involves significant use of electronic devices for monitoring, tracking, and alert systems. The energy consumption of these devices, along with the potential electronic waste from outdated or damaged equipment, could be considerable. The environmental impact must be carefully evaluated, with a focus on the lifecycle of the devices used, including their manufacturing, operation, and disposal stages.

Efforts to minimize the environmental footprint of the digital care pathway could include using energy-efficient devices, promoting recycling programs for electronic waste, and integrating renewable energy sources where possible. In addition, reducing the need for physical travel through remote monitoring and virtual consultations can help lower the overall carbon footprint.^13^ The effectiveness of these efforts would need to be assessed based on the specific measures implemented.

*Economic sustainability.* The proposed digital care pathway is likely to be cost-effective in the long run. By reducing the need for frequent in-person consultations and hospital visits, it can lower travel costs for patients and decrease the workload on healthcare professionals.^13,14^ This can lead to savings on salary costs and operational expenses. Moreover, improved seizure management and patient monitoring can potentially reduce emergency room visits and hospital admissions, further lowering healthcare costs.

The financial benefits include reduced costs associated with travel, hospital admissions, and emergency care due to better-managed epilepsy care. Moreover, there could be savings on staffing costs as remote monitoring and automation reduce the need for some routine in-person services. However, the initial investment in technology infrastructure, training for healthcare professionals, and ongoing maintenance costs could be seen as potential financial burdens.

*Social sustainability.* The digital care pathway can improve accessibility by providing remote monitoring and virtual consultations, making care more accessible to patients in remote or underserved areas.^13,42^ However, it also necessitates ensuring that patients have access to the required technology and possess sufficient digital literacy to use it effectively. Tailored educational programs and support services can help mitigate the risk of digital exclusion.

The digital care pathway can enhance job satisfaction by reducing routine administrative tasks and allowing healthcare professionals to focus more on patient care and complex decision-making. However, there may be concerns about the learning efforts associated with new technologies and the potential for increased screen time, which could lead to stress. Proper training and support systems can help to reduce these concerns. Finally, digital care pathways impact community activity, patient autonomy, and safety.^43^

Further discussion was done for evaluation of integration of the proposed AI-augmented framework into the digital care pathway including its usability and feasibility.

*Integration.* The proposed digital care pathway should be designed with interoperability in mind, allowing it to integrate seamlessly with existing healthcare systems and adapt to future technological advancements. This includes using standard communication protocols, ensuring data security and privacy, and fostering partnerships with technology providers. Integration with the current system requires careful planning and collaboration with Information Technology (IT) and clinical staff. Steps include conducting a thorough needs assessment, mapping out workflow changes, and ensuring compatibility with existing EHR and other healthcare systems. Training for healthcare professionals and ongoing technical support are essential to ensure a smooth transition and effective utilization of the new digital care pathway.

The unpredictability of seizures is a major issue which affects the quality of life for individuals with epilepsy. This can be mitigated by developing seizure prediction methods or identify seizures after they begin to manifest clinically and respond appropriately.^7^ Integration of seizure detection in DCPE has significant impact on the quality of life of people with epilepsy since it reduces seizure-related injuries. Intelligent seizure detection should be a priority in epilepsy management; however, it is advised not to rely solely on seizure detection devices.^7,8^

*Usability.* The digital pathway's design should prioritize flexibility and scalability to accommodate evolving healthcare practices. Its usability for both patients and healthcare professionals depend on continuous feedback, regular updates, and improvements based on user experience.^44–48^ Ensuring the pathway can adapt to new treatment protocols, technologies, and patient needs is crucial for its long-term sustainability and effectiveness.

Patients’ digital literacy levels vary, which can impact their ability to use the system effectively. Providing user-friendly interfaces and robust support for onboarding, education, and awareness can help mitigate this issue. Continuous technical support services, such as help desks, online tutorials, and one-on-one assistance, are essential for addressing patient concerns and troubleshooting issues in real time. The feasibility of providing these services depends on the institution's commitment to investing in comprehensive support structures.

Furthermore, the healthcare professionals added some additional comments for the proposed digital health care pathway:

*User-centered design.* The advice from the focus group discussion was to regularly incorporate feedback from both patients and healthcare professionals to continually improve the user experience and functionality of the digital care pathway. User-centered design principles should guide the development and enhancement of the system. This finding is aligned with the claim in the study by Herrera-Fortin et al.,^8^ where it is mentioned that guidelines for patient-centered designs are required as the epilepsy research community becomes more interested in seizure detection.

Healthcare professionals need to ensure that the platform is accessible to individuals with varying levels of digital literacy, including features like intuitive interfaces, clear instructions, and multilingual support. Patient engagement plays an important role in user-centered design in digital care pathway. Use of data from remote monitoring to create personalized care plans can be tailored to individual patient needs and preferences.^18^ Strategies can be personalized to keep patients engaged with their care, such as gamification, reminders, and regular health updates. Relevant data collection can assist health professionals to drive continuous improvement initiatives, ensuring the pathway remains relevant and effective over time.

*Data security and privacy.* Integration of AI-augmented system into the digital care pathway is very sensitive, thus, ensuring the system complies with relevant data protection regulations (e.g., General Data Protection Regulation (GDPR) and Health Insurance Portability and Accountability Act (HIPAA)) to protect patient data is the necessity. Consequently, implementing the robust cybersecurity measures to prevent data breaches and unauthorized access is very important and regular security audits and updates are essential.

*Training and support.* Training and support are required for healthcare professionals and patients to ensure they can effectively use the system. This should include initial training sessions and ongoing educational resources. It can establish a responsive technical support system to address any issues that users might encounter quickly. The availability of skilled IT staff who can manage and support advanced AI systems is critical for the AI-augmented digital care pathway. Currently, there is a growing pool of professionals with expertise in healthcare IT and AI, but specific training on the proposed system's nuances may be required. Comprehensive training programs for both healthcare professionals and technical support staff are necessary to ensure they can effectively use and maintain the system. Many institutions already have ongoing professional development programs, which could be expanded to include this training.

*Current technological infrastructure.* The current state of technological infrastructure in healthcare facilities is generally sufficient to support basic aspects of digital care pathways, such as remote monitoring and telemedicine. However, the specific requirements of the proposed system, such as advanced AI-augmented seizure tracking and personalized care planning, may necessitate upgrades or enhancements in existing systems. Ensuring that the new system can integrate seamlessly with existing EHRs and other digital platforms is crucial. Many healthcare facilities have already started adopting interoperable systems, which can facilitate the integration of the proposed pathway.

While technical support to ensure accessibility for patients within the proposed AI-integrated DCPE is feasible, it depends on several factors. These include the current state of technological infrastructure, availability of skilled personnel, effective training programs, patient digital literacy, financial resources, and regulatory compliance. With adequate planning and investment, healthcare facilities can provide the necessary technical support to make this innovative care pathway accessible and effective for patients.

## Conclusion

Digital tools can provide educational resources, self-management, and personalized care plans that empower individuals with epilepsy. These resources can help patients understand their condition better, track their symptoms, and actively participate in their treatment. Digital care pathways have the potential to revolutionize the delivery of care for epilepsy patients by increasing patient engagement and improving treatment outcomes. However, it is important to consider factors such as data privacy and security to ensure ethical and effective implementation of these technologies in epilepsy management.

EEG data is considered as effective data in diagnosing epilepsy, discuss various features and patterns that aid in identification, and explore the emerging role of AI in automating and improving the diagnostic process. Employing the power of EEG data analysis can enhance early detection and diagnosis of epilepsy, leading to more precise treatment strategies and improved outcomes for individuals living with this condition. Machine learning approaches offer promises in tracking epileptic seizures by analyzing patterns and features in EEG signals. The AI algorithms can assist in the automatic detection and classification of epileptic seizures by extracting relevant features from EEG signals using various time-domain, frequency-domain, and time-frequency analysis techniques.

Integrating seizure tracking and remote monitoring system in epileptic digital care pathway offers a platform for patient-centered care and shared decision-making to make the care pathway more sustainable. It provides appropriate and timely medication adherences and compliance. Moreover, personalized medicine and customized treatment in epilepsy management would be possible by understanding the unique EEG pattern of each patient. Early warning and prognosis can be possible by seizure tracking to take the preventive measures. Finally, long term monitoring could be possible to track changes in brain activity over the time for assessing the progress of epilepsy and the efficiency of the treatment.

Based on the focus group discussions, it was highlighted that:

It is better to start with the adults at the emergency and ICU department first. After the system is validated, it can be specialized for patients of different age groups, preferably above the age of 12 years old. In the future, the system can be used for patients with different age group and demographics. (expert 1, 2 & 3)

Therefore, the primary goal in future is to implement EEG-based seizure detection specifically for emergency situations, like prolonged seizures or status epilepticus, starting from the emergency room to ICU. A critical theme is the need to minimize diagnosis time during severe epileptic events, emphasizing rapid detection and treatment initiation. Furthermore, there are challenges related to balancing the three main sustainability pillars: social, economic, and environmental for and AI-augmented DCPE. Future studies will focus on addressing these challenges and incorporating the technological pillar into the sustainability framework for the AI-augmented digital care pathway.

The main limitation of this study was lack of the recent dataset. For this study, an EEG dataset collected in Department of Epileptology, University Hospital Bonn was used. This sample dataset was used for the purpose of exploring and assessing the feasibility, usability, and sustainability of the integration of AI-augmented seizure detection in digital care pathway from healthcare professionals’ perspective. Furthermore, the questionnaire used for focus group discussion was pilot-tested, but it could be validated further for future use. Finally, a suitable sustainability farm work for measuring sustainability of specific AI-augmented digital care pathway is missing.

## Supplemental Material

sj-docx-1-dhj-10.1177_20552076241287356 - Supplemental material for A sustainable artificial-intelligence-augmented digital care pathway for epilepsy: Automating seizure tracking based on electroencephalogram data using artificial intelligence Supplemental material, sj-docx-1-dhj-10.1177_20552076241287356 for A sustainable artificial-intelligence-augmented digital care pathway for epilepsy: Automating seizure tracking based on electroencephalogram data using artificial intelligence by Pantea Keikhosrokiani, Minna Isomursu, Johanna Uusimaa and Jukka Kortelainen in DIGITAL HEALTH

sj-pdf-2-dhj-10.1177_20552076241287356 - Supplemental material for A sustainable artificial-intelligence-augmented digital care pathway for epilepsy: Automating seizure tracking based on electroencephalogram data using artificial intelligence Supplemental material, sj-pdf-2-dhj-10.1177_20552076241287356 for A sustainable artificial-intelligence-augmented digital care pathway for epilepsy: Automating seizure tracking based on electroencephalogram data using artificial intelligence by Pantea Keikhosrokiani, Minna Isomursu, Johanna Uusimaa and Jukka Kortelainen in DIGITAL HEALTH
