# Supplementary material for: A sustainable artificial-intelligence-augmented digital care pathway for epilepsy: Automating seizure tracking based on electroencephalogram data using artificial intelligence
Source: Digit Health. 2024 Oct 7;10:20552076241287356. doi: 10.1177/20552076241287356 (PMC11459578; doi:10.1177/20552076241287356)
Supplement: sj-docx-1-dhj-10.1177_20552076241287356 - Supplemental material for A sustainable artificial-intelligence-augmented digital care pathway for epilepsy: Automating seizure tracking based on electroencephalogram data using artificial intelligence [file sj-docx-1-dhj-10.1177_20552076241287356.docx]

| **System Requirement Analysis for EEG-based Seizure Detection System Integration in Epileptic Digital Care Pathway** |
| --- |
| **Description:**  EEG-based Seizure Detection System (AI-EpiDigi) aims to monitor individuals with epilepsy remotely to track and classify their seizures from time to time. Suitable wearables will be used to collect EEG data from the patients and the data will be transferred to AI-EpiDigi cloud for analytical purposes. A machine learning algorithm with high accuracy will be used for the proper seizure detection and classification. The results with summary report will be displayed in  the healthcare professional’s analytical dashboard for further action. Figure 1 shows the general overview of AI- EpiDigi, which will be added into the Epileptic Digital Care Pathway.  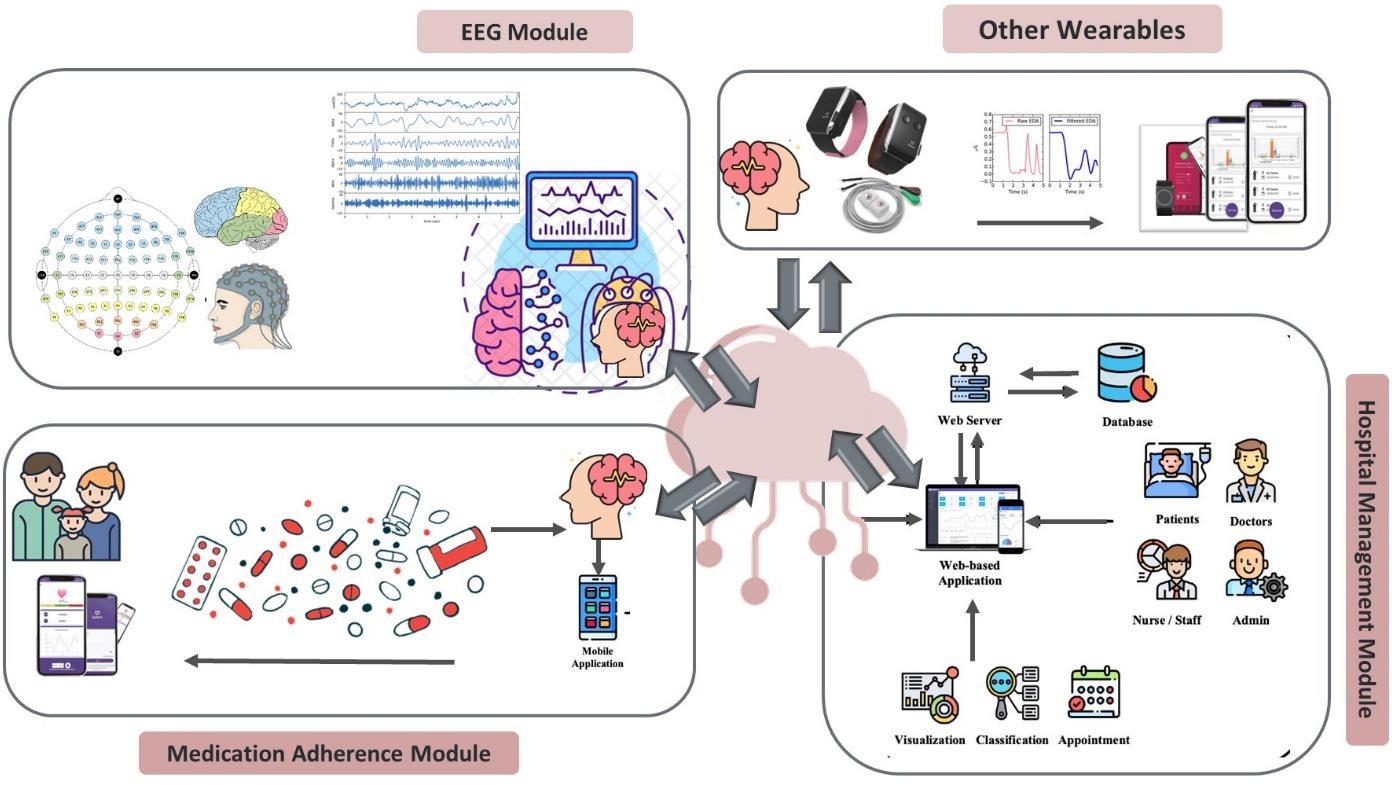  Figure 1. Overview of AI-EpiDigi |
|  |
| **Needs Assessment** |
| - **Objectives & Stakeholders:**   - What are the primary goals of EEG-based seizure detection in epileptic digital care pathway?   - Which phase the EEG-based seizure detection can be fitted into the current digital care pathway to accurately detect epilepsy seizures using EEG and wearable data?   - Who are the potential stakeholders and users of this digital care pathway (patients, doctors, caregivers, healthcare institutions, technical staff, etc.)? |
| **Requirement Elicitation** |
| - **Functional Requirements:**   - What the EEG-based seizure detection system should do (e.g., data collection, processing, seizure detection, alerting mechanisms)?   - What features do you believe are essential for an EEG-based seizure detection system?   - Are there any specific functions you would like the wearable device to perform?   - In case of detected seizures, who should be alerted (e.g., patient, caregiver, doctor)? (Alert and Notification System)   - What mode of alerts do you prefer (e.g., SMS, app notification, email)? (Alert and Notification System) - **Non-Functional Requirements:** |

| - What do you need to know about the seizures? (e.g., types of the seizures, seizure or not, duration of the seizure, etc.) - What type of training or support materials would be helpful for new users of this system? - Would you prefer online tutorials, written manuals, or in-person training sessions? - **Regulatory and Compliance Needs:**   - What are the necessary medical and data regulations the system must comply with (e.g., FDA, HIPAA, GDPR)? - **Usability and Design:**   - What are your preferences regarding the size and design of the wearable device?   - How important is the aesthetic appeal of the wearable device to you (scale of 1-5)? - **Data Management and Security**   - What are your concerns regarding data security and privacy for EEG-based Seizure Detection System?   - How would you like to access and review the data collected by the EEG-based Seizure Detection System? - **Cost and Accessibility**   - What are your expectations regarding the cost of the system?   - How important is it for this system to be covered by insurance or healthcare providers (scale of 1-5)? |
| --- |
| **Requirement Analysis and Prioritization** |
| - **Feasibility Study:**   - How the idea is feasible economically and operationally? - **Risk Analysis:**   - What are the potential risks and mitigation strategies? - **Requirement Prioritization:**   - What are the requirements of the EEG-based seizure detection system based on importance and urgency? |
| **Sustainability** |
| **Environmental Sustainability**   - How do you think about the environmental impact of implementing the proposed digital care pathway in terms of energy consumption and electronic waste? - How would you rate the efforts made to minimize the environmental footprint of the digital care pathway? toxicity   **Economic Sustainability**   - Do you believe the proposed digital care pathway is cost-effective in the long run? Please explain your reasoning. Save Salary cost of HCP and travelling for patient - What are the anticipated financial benefits or burdens of adopting this digital pathway for your healthcare facility?   **Social Sustainability**   - How does the proposed digital care pathway address accessibility and equity issues (digital literacy can have negative effects if cannot use it or positive effect …..) within patient care?   In what ways does the proposed digital care pathway contribute to or reduce job satisfaction among healthcare professionals? HCP |
| **Usability** |
| Considering sustainability, how well does the digital pathway support long-term adaptability to changes in healthcare practices? Mainly for the accessibility side for patients and HCP if they feel is beneficial and add value |
| **Integration** |
| How would you evaluate the potential of the proposed digital care pathway to remain compatible with future  technological advancements in healthcare. How to integrate it with the current system? |

|  |
| --- |
| Would you like to provide any additional comments or suggestions? |

Thank you for your cooperation.
